# Supplementary material for: Resolving the full spectrum of human genome variation using Linked-Reads
Source: Genome Res. 2019 Apr;29(4):635–45. doi: 10.1101/gr.234443.118 (PMC6442396; doi:10.1101/gr.234443.118)
Supplement: Supplemental Material [file supp_gr.234443.118_Supplemental_longranger-2.2.zip › longranger-2.2.tar.bz2!/lr2.2-public/lib/stan/cmdstan/stan/doc/api/html/math_2matrix_2trace__gen__inv__quad__form__ldlt_8hpp_source.html]

Stan: src/stan/math/matrix/trace\_gen\_inv\_quad\_form\_ldlt.hpp Source File


|  |  |
| --- | --- |
|  | Stan  2.5.0  probability, sampling & optimization |


- Main Page
- Related Pages
- Namespaces
- Classes
- Files

- File List
- File Members

All Classes Namespaces Files Functions Variables Typedefs Enumerations Enumerator Friends Macros Pages

- src
- stan
- math
- matrix

trace\_gen\_inv\_quad\_form\_ldlt.hpp

Go to the documentation of this file.

1 #ifndef STAN\_\_MATH\_\_MATRIX\_\_TRACE\_GEN\_INV\_QUAD\_FORM\_LDLT\_HPP

2 #define STAN\_\_MATH\_\_MATRIX\_\_TRACE\_GEN\_INV\_QUAD\_FORM\_LDLT\_HPP

3

4 #include <stan/math/matrix/Eigen.hpp>

5 #include <stan/math/matrix/LDLT\_factor.hpp>

6 #include <stan/math/error\_handling/matrix/check\_multiplicable.hpp>

7 #include <stan/math/error\_handling/matrix/check\_square.hpp>

8 #include <stan/math/matrix/mdivide\_left\_ldlt.hpp>

9 #include <stan/math/matrix/trace.hpp>

10 #include <stan/math/matrix/transpose.hpp>

11 #include <stan/math/matrix/multiply.hpp>

12

13 namespace stan {

14  namespace math {

15

16  /\*

17  \* Compute the trace of an inverse quadratic form. I.E., this computes

18  \* trace(D B^T A^-1 B)

19  \* where D is a square matrix and the LDLT\_factor of A is provided.

20  \*/

21  template <typename T1, typename T2, typename T3,

22  int R1,int C1,int R2,int C2,int R3,int C3>

23  inline typename

24  boost::enable\_if\_c<!stan::is\_var<T1>::value &&

25  !stan::is\_var<T2>::value &&

26  !stan::is\_var<T3>::value,

27  typename boost::math::tools::promote\_args<T1,T2,T3>::type>::type

28  trace\_gen\_inv\_quad\_form\_ldlt(const Eigen::Matrix<T1,R1,C1> &D,

29  const stan::math::LDLT\_factor<T2,R2,C2> &A,

30  const Eigen::Matrix<T3,R3,C3> &B) {

31

32  stan::math::check\_square("trace\_gen\_inv\_quad\_form\_ldlt(%1%)",D,"D",

33  (double\*)0);

34  stan::math::check\_multiplicable("trace\_gen\_inv\_quad\_form\_ldlt(%1%)",A,"A",

35  B,"B",(double\*)0);

36  stan::math::check\_multiplicable("trace\_gen\_inv\_quad\_form\_ldlt(%1%)",B,"B",

37  D,"D",(double\*)0);

38

39  return trace(multiply(multiply(D,transpose(B)),mdivide\_left\_ldlt(A,B)));

40  }

41

42  }

43 }

44 #endif

mdivide\_left\_ldlt.hpp

check\_multiplicable.hpp

stan::is\_var

**Definition:** traits.hpp:421

stan::math::mdivide\_left\_ldlt

Eigen::Matrix< typename boost::math::tools::promote\_args< T1, T2 >::type, R1, C2 > mdivide\_left\_ldlt(const stan::math::LDLT\_factor< T1, R1, C1 > &A, const Eigen::Matrix< T2, R2, C2 > &b)

Returns the solution of the system Ax=b given an LDLT\_factor of A.

**Definition:** mdivide\_left\_ldlt.hpp:24

transpose.hpp

trace.hpp

stan::math::check\_multiplicable

bool check\_multiplicable(const char \*function, const T1 &y1, const char \*name1, const T2 &y2, const char \*name2, T\_result \*result)

**Definition:** check\_multiplicable.hpp:15

stan::math::transpose

Eigen::Matrix< T, C, R > transpose(const Eigen::Matrix< T, R, C > &m)

**Definition:** transpose.hpp:12

stan::math::trace\_gen\_inv\_quad\_form\_ldlt

boost::enable\_if\_c<!stan::is\_var< T1 >::value &&!stan::is\_var< T2 >::value &&!stan::is\_var< T3 >::value, typename boost::math::tools::promote\_args< T1, T2, T3 >::type >::type trace\_gen\_inv\_quad\_form\_ldlt(const Eigen::Matrix< T1, R1, C1 > &D, const stan::math::LDLT\_factor< T2, R2, C2 > &A, const Eigen::Matrix< T3, R3, C3 > &B)

**Definition:** trace\_gen\_inv\_quad\_form\_ldlt.hpp:28

stan::math::LDLT\_factor

**Definition:** LDLT\_factor.hpp:16

Eigen.hpp

stan::math::trace

T trace(const Eigen::Matrix< T, Eigen::Dynamic, Eigen::Dynamic > &m)

Returns the trace of the specified matrix.

**Definition:** trace.hpp:20

stan::math::multiply

boost::enable\_if\_c< boost::is\_arithmetic< T >::value, Eigen::Matrix< double, R, C > >::type multiply(const Eigen::Matrix< double, R, C > &m, T c)

Return specified matrix multiplied by specified scalar.

**Definition:** multiply.hpp:25

multiply.hpp

stan::math::check\_square

bool check\_square(const char \*function, const Eigen::Matrix< T\_y, Eigen::Dynamic, Eigen::Dynamic > &y, const char \*name, T\_result \*result)

Return true if the specified matrix is square.

**Definition:** check\_square.hpp:24

LDLT\_factor.hpp

check\_square.hpp


---

[ Stan Home Page ]

*© 2011–2014,
Stan Development Team.*
